# A Comprehensive, Quantitative, and Genome-Wide Model of Translation

**DOI:** 10.1371/journal.pcbi.1000865

**Published:** 2010-07-29

**Authors:** Marlena Siwiak, Piotr Zielenkiewicz

**Affiliations:** 1 Institute of Biochemistry and Biophysics, Polish Academy of Sciences, Warsaw, Poland; 2 Laboratory of Plant Molecular Biology, Faculty of Biology, Warsaw University, Warsaw, Poland; Weizmann Institute of Science, Israel

## Abstract

Translation is still poorly characterised at the level of individual proteins and its role in regulation of gene expression has been constantly underestimated. To better understand the process of protein synthesis we developed a comprehensive and quantitative model of translation, characterising protein synthesis separately for individual genes. The main advantage of the model is that basing it on only a few datasets and general assumptions allows the calculation of many important translational parameters, which are extremely difficult to measure experimentally. In the model, each gene is attributed with a set of translational parameters, namely the absolute number of transcripts, ribosome density, mean codon translation time, total transcript translation time, total time required for translation initiation and elongation, translation initiation rate, mean mRNA lifetime, and absolute number of proteins produced by gene transcripts. Most parameters were calculated based on only one experimental dataset of genome-wide ribosome profiling. The model was implemented in *Saccharomyces cerevisiae*, and its results were compared with available data, yielding reasonably good correlations. The calculated coefficients were used to perform a global analysis of translation in yeast, revealing some interesting aspects of the process. We have shown that two commonly used measures of translation efficiency – ribosome density and number of protein molecules produced – are affected by two distinct factors. High values of both measures are caused, i.a., by very short times of translation initiation, however, the origins of initiation time reduction are completely different in both cases. The model is universal and can be applied to any organism, if the necessary input data are available. The model allows us to better integrate transcriptomic and proteomic data. A few other possibilities of the model utilisation are discussed concerning the example of the yeast system.

## Author Summary

Translation is the production of proteins by decoding mRNA produced in transcription, and is a part of the overall process of gene expression. Although the general theoretical background of translation is known, the process is still poorly characterised at the level of individual proteins. In particular, the quantitative parameters of translation, such as time required to complete it or the number of protein molecules produced from a transcript during its lifetime, are extremely difficult to measure experimentally. To overcome this problem, we developed a computational model that, on the basis of only few datasets and general assumptions, measures quantitatively the translational activity at the level of individual genes. We discussed it concerning the example of the yeast system; however, it can be applied to any organism of known genome. We used the obtained results to study the general characteristics of the yeast translational system, revealing the diversity of strategies of gene expression regulation. We exemplified and discussed other possible ways of model utilisation, as it may help in examining protein-protein interactions, metabolic pathways, gene annotation, ribosome queueing, protein folding, and translation initiation. It also may be crucial for better integration of cell-wide, high-throughput experiments.

## Introduction

The rate of translation differs for individual proteins, reflecting both the intrinsic capability of an mRNA molecule to be translated and the environmental factors affecting the efficiency of the translation process. The first is well characterised in other studies [1]–[3] that discuss mRNA features responsible for the regulation of translation (e.g., length of the 5′ UTR, presence and location of 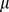
ORFs, type and number of initiation codons, sequence context around the initiation codon, presence and location of mRNA secondary structure elements, codon usage, mRNA stability, and posttranscriptional modifications). However, the second describes the features of the environment in which translation occurs, namely the amounts of particular mRNA transcripts in a cell, the accessibility of the translation machinery elements required to initiate and accomplish protein synthesis (such as free ribosomes, tRNAs, and elongation factors), as well as growth conditions, which have been proven to evoke gene-specific translational control [4].

Although the general theoretical background of translation is known, the process of protein synthesis is still poorly characterised at the level of individual proteins. Experimental determination of absolute translation rates (i.e., in time units) is a tremendous task and we are not aware of any such research. Even though the factors specified above have been studied separately for some proteins, little is known about the extent to which they affect the process and how they cooperate to keep the synthesis rate at the required level. Another strategy to examine translation activity is to integrate genome-wide expression datasets from different sources [5]–[8]. However, it was shown [9] that these datasets cannot be used to predict translation rates at the level of individual proteins, as they suffer from large random errors and systematic shifts in reported values.

In practice, upon the development of techniques to examine transcriptome data experimentally (microarrays, Northern blotting, RNA-seq, etc.), the mRNA concentration has become a broadly used measure of protein abundance. Nevertheless, recent research indicates that there is only a partial correlation between mRNA and protein abundances [10]–[16]. It was shown that the mRNA transcription level can explain only 20–40% of the observed amounts of proteins [17], [18], which leads to conclusion that the role of translation in regulation of gene expression has been constantly underestimated. Thus, a deeper insight into the process of translation is required to better integrate transcriptomic and proteomic data [19]–[21].

In this study, we developed a model to measure the absolute, translational activity at the level of individual genes. The model was implemented in *Saccharomyces cerevisiae*, however, it can be used to study translation in any other organism of known genome, but only if the following data are available: (i) a dataset of mRNA relative abundance and ribosome footprints; (ii) tRNAs decoding specificities; (iii) average cell volume; (iv) average number of active ribosomes in a cell; (v) average number of mRNA transcripts in a cell; and (vi) a dataset of mRNA half-lives (optionally).

In our calculations for the yeast system the first dataset came from one genome-wide experiment provided by Ingolia et al. [22], quantifying simultaneously mRNA abundance and ribosome footprints by means of deep sequencing. This method is thought to provide a far more precise measurement of transcript levels than other hybridisation or sequence-based approaches [23]. Based on this dataset, we determined the absolute time of translation, in SI units, for individual genes. The time is the sum of the time required to accomplish two main steps of protein translation: initiation and elongation. Analysing the initiation or elongation time alone provides quantitative information on the extent of translation regulation at these two steps separately. Moreover, by introducing mRNA concentrations into the model, one can calculate the relative rate of translation initiation, which does not depend on the transcriptional level of a corresponding gene. Assuming identical conditions for all mRNAs in the cell (i.e., equal amounts of available ribosomes, elongation factors, tRNAs, etc.), the measure will reflect the mRNA's intrinsic ability (in relation to other analysed mRNAs) to regulate the efficiency of translation initiation. Such a deep insight into the process of initiation is particularly important, as this step of protein synthesis is thought to be the main and rate-limiting target for translational control [24]. Furthermore, by combining our results with a dataset on mRNA stability [25], we calculated the absolute amounts of protein produced from each transcript during its lifespan.

We compared our results with direct experimental studies measuring the mRNA and protein levels of chosen genes. Good correlation with most of the experimental data was observed, and calculated mRNA and protein abundances did not differ significantly from those reported *in vivo*. In addition, other calculated parameters of translation, such as the overall rate of protein synthesis, were in agreement with earlier reports.

The calculated translational parameters were also used to study the general characteristics of the yeast translational system, revealing the diversity of strategies of gene expression regulation. For instance, we showed that two commonly used measures of translation efficiency – ribosome density and number of protein molecules produced – are affected by two distinct factors. We observed strong negative correlations between values of both measures and translation initiation time, however, the origins of initiation time reduction for most efficient transcripts are completely different. In case of elevated ribosome density, short initiation is caused mostly by mRNA instristic capability of being translated discussed at the beginning of this section. Contrary, in case of high number of protein molecules produced, short initiation is caused primarily by elevated mRNA concetrations.

Finally, we exemplified and discussed other possible ways of model utilisation, as the model may be of considerable help in examining gene expression regulation, protein-protein interactions, metabolic pathways, gene annotation, ribosome queuing, protein folding, and translation initiation. Additionally, the model provides an overall and quantitative picture of the translation process, crucial for better integration of transcriptomic and proteomic data from high-throughput experiments.

## Results

The following translational parameters were attributed to the yeast genes (for derivation, see the Materials and Methods): 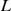, length of the transcript coding sequence (CDS) in codons; 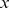, absolute number of transcripts in a yeast cell; 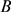, total amount of protein molecules produced from transcripts of particular type; 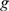, ribosome density in number of ribosomes attached to a transcript per 100 codons; 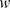, the absolute number of ribosomes on a transcript; 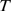, total time of translation of one protein molecule from a given transcript; 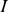, total time required for translation initiation; 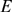, total time required for translation elongation; 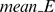, mean time required for elongation of one codon of a transcript; 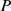, translation initiation frequency; 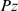, relative rate of binding of free ribosomes to the 5′ end of a transcript, proportional to the concentration of the transcript; 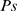, relative rate of successful accomplishments of initiation once the ribosome-mRNA complex is formed (the obtained values of the parameter 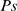 ranged from 3.4e-4 to 65.9. For clarity, we decided to normalise them by the maximal reported value of 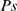 obtained for the gene YLL040C. The normalised values of 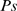 range from 0 to 1 and allow more intuitive comparison); 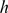, estimated half-life of a transcript; and 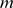, estimated mean lifetime of a transcript. Parameters 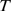, 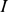, 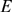, 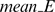, 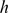, and 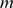 are given in SI units.

We managed to attribute quantitative measures of translation to the majority of 4648 transcripts from the initial dataset. Four transcripts were rejected at the beginning of processing, as ribosome footprints were not observed on them. Further, 23 transcript had unrealistic, elevated 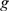 values (i.e., 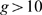). Assuming, that a ribosome covers ten codons, a transcript CDS built of 100 codons cannot contain more than ten ribosomes. Eventually, we eliminate transcripts at which queuing of the ribosomes may occur. Our simulation program yielded 130 transcripts suspected of queuing, plus 21 for which translation at the 5′ end is so slow that the first attached ribosome prevents the attachment of the following ribosomes. Further calculations were performed for the most relevant transcripts, i.e., the remaining 4470 yeast genes, without ribosome queuing.

The values of parameters 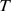, 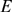, 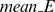, 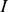, 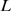, 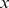, 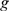, 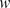, 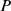, 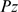, and normalised 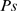 were determined for all 4470 transcripts in the dataset, of which 4192 could also be attributed with additional parameters 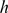, 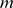, and 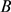. The calculation of all parameters except 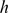, 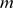, and 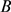 was entirely based on the results from one high-throughput experiment. Parameters 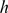, 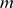, and 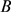 engaged one additional dataset of mRNA relative half-lives. The general characteristics of the parameters are specified in Table 1. The values of parameters for individual genes are provided in Supplementary Table S1.

**Table 1:** The translational parameters calculated in the model.

par	mean	median	sd	min	max	description
L	513.3	430.5	365.2	37	4911	Length of the transcript CDS in codons.
x	7.8	2.7	28.9	0.140	591.3	Absolute number of transcripts in a yeast cell.
B	1.0e+4	677	7.7e+4	0.650	2.4e+6	Total amount of protein molecules produced from transcripts of a particular type.
g	1.1	0.8	0.9	0.003	6.6	Ribosome density in number of ribosomes attached to a transcript per 100 codons.
w	5.6	3.1	7.3	0.010	142	The absolute number of ribosomes on a transcript.
P	5.3e-5	3.6e-5	5.4e-5	1.5e-7	6.2e-4	The translation initiation frequency (the inverse of I).
Pz	2.2e-4	7.6e-5	8.0e-4	3.8e-6	1.6e-2	The relative rate of binding of free ribosomes to the 5′ end of a transcript.
Ps	1.6e-2	6.4e-3	2.9e-2	5.2e-6	4.3e-1	The relative rate of a successful accomplishment of initiation once the ribosome-mRNA complex is formed, normalised by the maximal observed value of Ps, reported for gene YLL040C.
T	2:50	2:20	3:23	0:06	113:08	Total time of translation of one protein molecule from a given transcript (min:sec).
I	0:54	0:28	3:06	0:02	111:54	Total time required for translation initiation (min:sec).
E	1:56	1:36	1:24	0:04	17:54	Total time required for translation elongation of a transcript (min:sec).
mean_E	0.224	0.229	0.031	0.098	0.360	Mean time required for elongation of one codon of a transcript (sec).
h	2:45:51	1:31:44	3:59:18	0:00:19	42:27:31	Estimated half-life of a transcript (h:min:sec).
m	3:59:16	2:12:20	5:45:13	0:00:27	61:15:18	Estimated mean life-time of a transcript (h:min:sec).

Column descriptions: (1) name of the parameter; (2) mean value; (3) median value; (4) standard deviation; (5) minimal observed value; (6) maximal observed value; and (7) parameter description. For all parameters, except 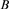, 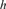, and 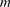, the columns 2, 3, 4, 5, and 6 were calculated over the entire dataset of 4,470 yeast genes. For parameters 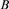, 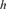, and 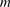 the columns 2, 3, 4, 5, and 6 were calculated over the set of 4,192 genes.

**Figure 1:**
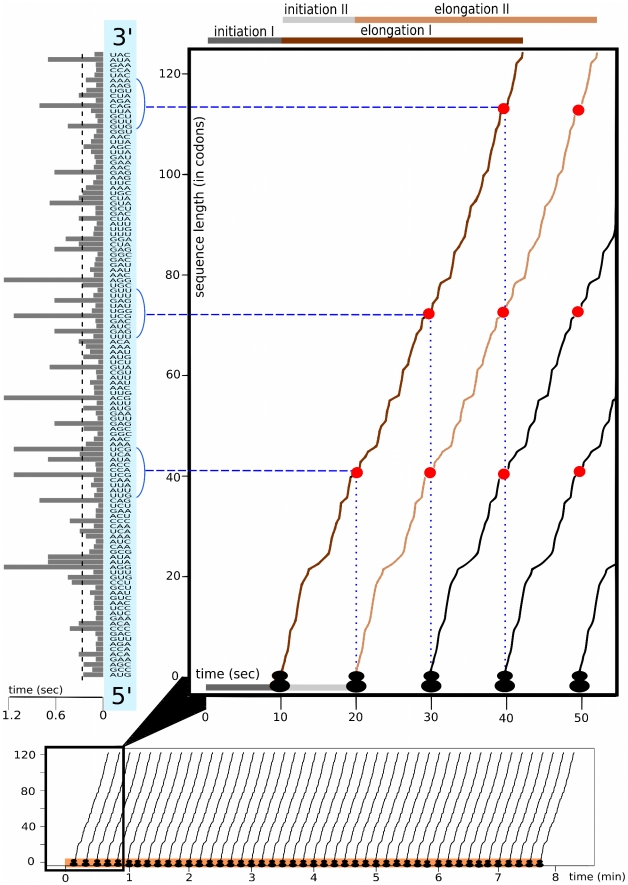
Translation model of YJL173C. The bottom plot shows all of the translation initiation events during the mean lifetime of one mRNA molecule. Translation initiations are marked with ribosome-shaped symbols. The orange line indicates the mean lifetime of YJL173C mRNA. The broken curves' slope depicts the rate of polypeptide chain growth measured at particular codons. The number of curves indicates the number of protein molecules (here 46) produced from one mRNA during its lifetime. The top-right plot shows, in magnitude, the translation of the first protein molecule (darkbrown curve). The time is measured since the transcript becomes accessible to the translation machinery. The first seconds are spent on translation initiation; elongation begins after about 10 sec. Red dots mark ribosome positions in time (dotted blue lines) and space (dashed blue lines) when the following ribosomes attach to the mRNA molecule. The histogram on the left shows the mean translation times of particular codons of the YJL173C sequence. The dashed black line is the mean time of translation of one codon of the YJL173C mRNA sequence.

The calculated parameters allow to study the process of translation at the level of individual genes. Figure 1 depicts the translation process in time on the example of protein YJL173C, a highly conserved subunit of Replication Protein A (RPA). Similar schematics may be constructed for the majority of yeast genes.

### Correlations with existing data

**Figure 2:**
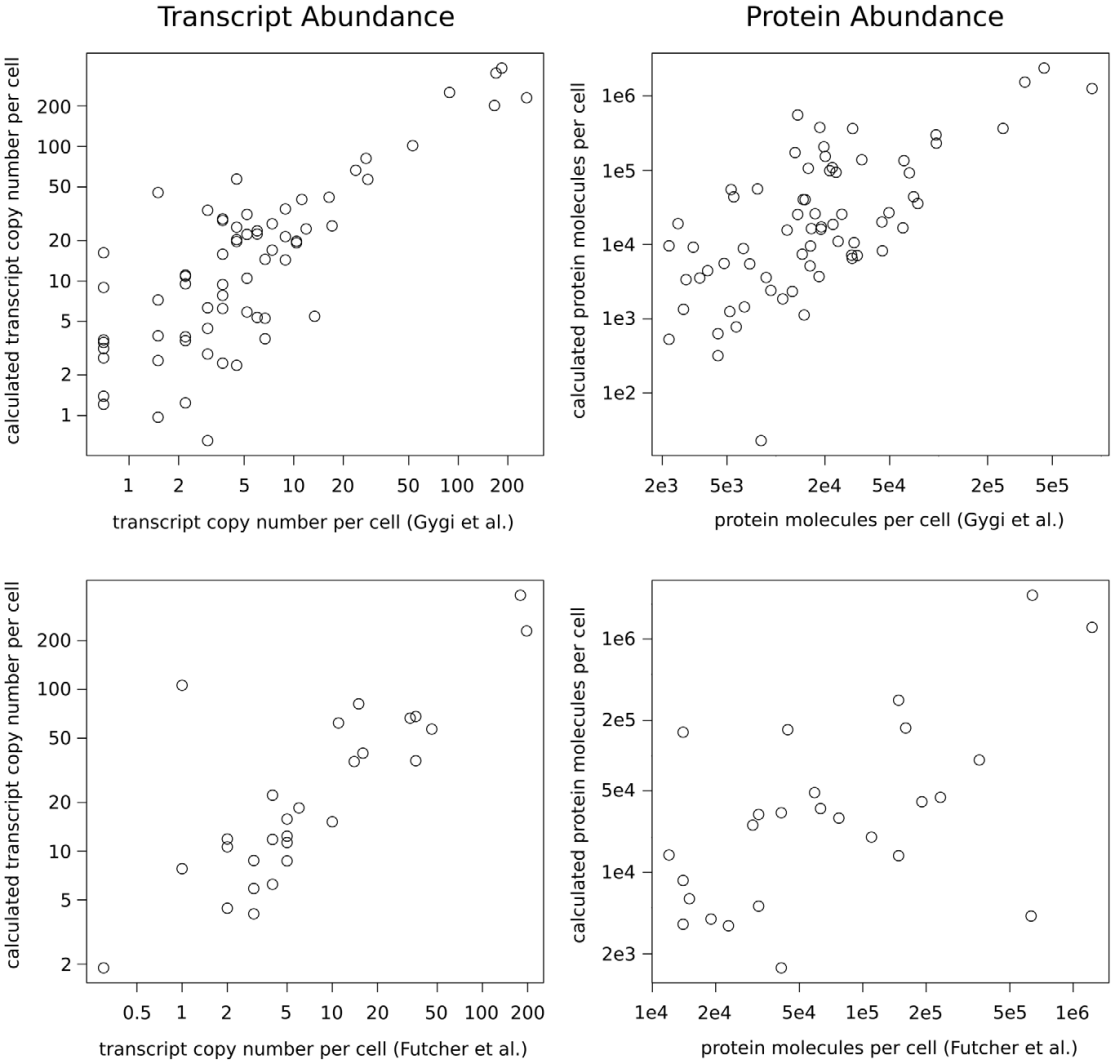
Model results vs experimental studies. The plots show the comparison of model parameters 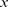 (left) and 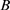 (right) with experimentally determined mRNA and protein abundances by two independent studies [13], [26]. The axes were log transformed. Calculated 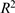 values are presented in Table 2. The distribution of the log-fold differences of the mRNA and protein concentrations reported by the model and reference studies are presented in Supplementary Figure S1.

Next, we checked if our calculations were in agreement with published data on protein and mRNA abundances. We compared our results with two previously published studies that provide information on transcript and protein copy number for numerous *S. cerevisiae* genes [13], [26], by performing linear regression through the origin on the log-transformed values. The adjusted 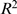 values, as well as the corresponding regression coefficients, were calculated for six pairs of datasets and the results are presented in Table 2. Scatter plots are presented in Figure 2. The results show that our model explains 84% of the variability in mRNA abundance and 97% of the variability in protein abundance reported by experimental studies. Such 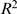 values are reasonably good, taking into account the differences in the particular yeast strains and laboratory protocols used, as well as the fact that our calculations are based on a few simplifications that can disrupt the final outcome. Moreover, 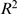 values reported for our model do not stand out from those calculated for comparisons of two experimental datasets with each other, suggesting that the observed differences constitute the internal variability of the system, not a methodology error. To measure if our results suffer from systematic shift, we calculated the fold difference values for transcript and protein abundance comparisons with two experimental datasets (see Supplementary Figure S1). In general, our calculations slightly overestimate the transcript copy number and underestimate the protein copy number, in relation to published data. This is mainly caused by the assumption we made: that one yeast cell contains, on average, 36,000 transcripts. The transcript copy number used in both reference studies is originally taken from older research [27], which quantified the relative mRNA concentrations and transformed them into absolute copy number, assuming 15,000 as the total number of transcripts per cell. This estimation seems inadequate to us in the light of current discoveries, which are explained in the Materials and Methods.

**Table 2:** Model determined mRNA and protein abundances versus experimental studies.

compared datasets	mRNA abundances			protein abundances		
	common genes	adj. 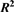	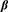	common genes	adj. 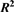	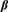
our dataset vs Gygi et al.	67	0.84	1.25	69	0.97	1.01
our dataset vs Futcher et al.	28	0.84	1.24	26	0.98	0.92
Gygi et al. vs Futcher et al.	25	0.97	1.04	27	0.99	0.91
our dataset vs Holstege et al.	3769	0.30	0.75			

The comparison of mRNA and protein abundances obtained in the model (reflected by parameters 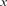 and 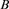) with values reported by three independent experimental studies [13], [26], [29]. We performed a simple linear regression through the origin on the log-transformed values. Column descriptions: (common genes), number of common genes in two compared datasets; (adj. 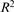), adjusted 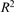 values for the linear regression model; and (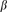), regression coefficient. The third row is the comparison of the two experimental studies with each other. All coefficients were statistically significant (F-statistic p-values 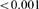).

**Figure 3:**
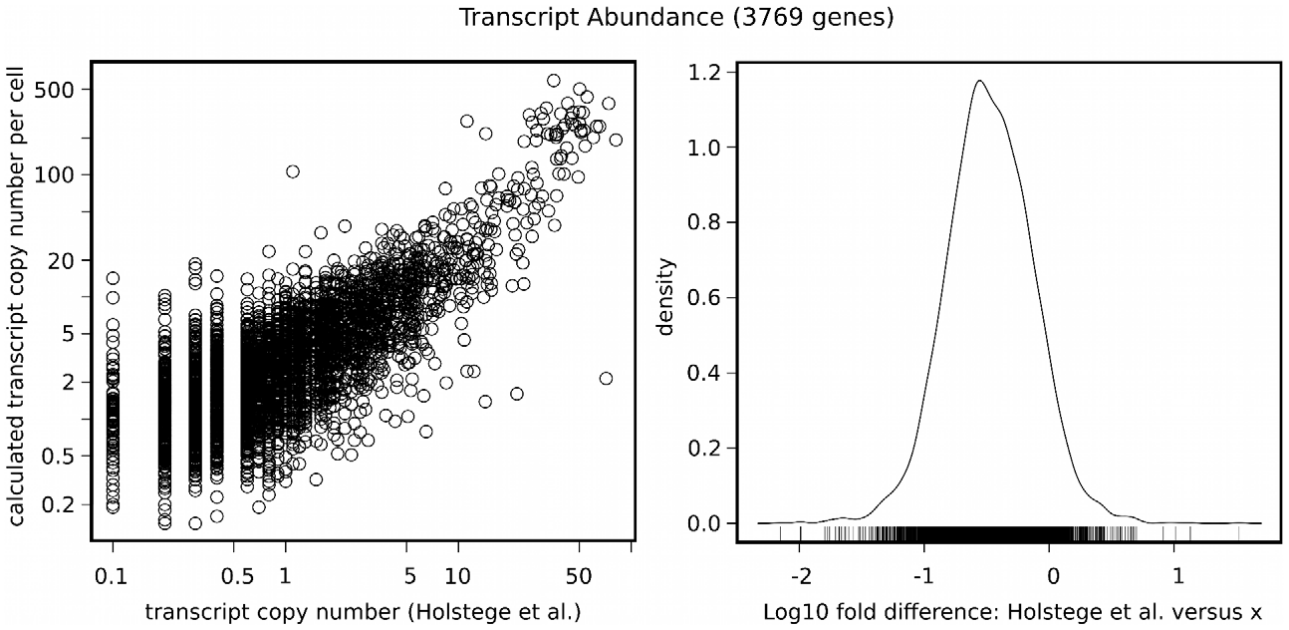
Calculated transcript abundance vs experimental studies. Left plot: the comparison of model parameter 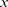 with mRNA abundances determined by high-density oligonucleotide array (HDA) experiment [29]. The axes were log transformed. Calculated 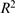 value for the comparison is presented in Table 2. Right plot: distribution of the log-fold differences of the mRNA concentrations reported by the model and reference study.

Transcript copy number is also problematic due to the wide discrepancies in mRNA levels reported by different studies [28]. Above mentioned mRNA concentration dataset [27] was obtained in a serial analysis of gene expression (SAGE) experiment and it is likely that such concentration estimates have low precision for low abundance mRNAs [13], [26]. On the other hand, it is hypothesized that SAGE is more accurate for abundant mRNAs when compared with other widely used technique: high-density oligonucleotide arrays (HDA) [26], [28]. Thus, we decided to compare mRNA concentrations calculated in our model with results obtained in genome-wide HDA experiment [29]. We performed linear regression through the origin on log-transformed data on mRNA abundance for 3769 genes. Scatter plot and the distribution of fold difference values are presented in Figure 3. The obtained adjusted 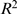 value was 0.30 (see Table 2), meaning that parameter 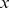 is able to explain only one third of the variability in mRNA abundance reported by this experiment [29]. This discrepancy is probably caused again by the experimental error. Parameter 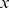 reflects mRNA concentration obtained by means of deep-sequencing, technique considered to be far more precise in measuring mRNA levels than other hybridisation or sequence-based approaches [23]. However, it is likely, that it is less precise for low abundance mRNAs, which may be seen in Figure S1 provided by Ingolia et al. [22]. This would explain why parameter 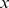 better describes variability in mRNA concentrations obtained from SAGE than HDA experiments.

In addition, we estimated that the cell-wide rate of translation for *S. cerevisiae* at 30

C is 5.5 amino acids (aa) per second, which corresponds to an average time of translation for one codon of 183 ms. This is in agreement with experimental studies, reporting rates of 8.8 aa/sec and 5.2 aa/sec for fast-growing and slow-growing yeast cells, respectively [30]. It is worth noting that the obtained value is also within the range reported for proteins from other organisms, namely 6 aa/sec for human apolipoprotein [31], 0.74 aa/sec for rabbit hemoglobin [32], 5 aa/sec for chick ovalbumin [33], and an average translation rate of 7.3 aa/sec in cockerel liver [34].

Furthermore, it is reported in independent studies that the total amount of protein in a yeast cell varies from 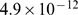 g [16] to 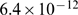 g [35]. Based on known protein sequences and the molecular mass of particular amino acids, we can calculate the mass of each yeast protein. By multiplying this by the protein copy number 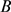 and summing over all expressed yeast proteins, we estimated that the total mass of proteins in a yeast cell is around 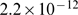 g. Although this number is smaller than values reported previously, it is still consistent taking into account the fact that we excluded from the calculations all transcripts with ribosome density 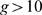, as our model cannot operate on such elevated values of this parameter. Most likely, 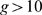 results in very high level of translation, meaning that excluded transcripts would have large 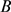 values, if they could be counted by our model. Thus, excluding these transcripts strongly affects the final mass of proteins in a yeast cell, diminishing it noticeably. Moreover, we must not forget that calculated values of the parameter 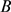 reflect only the total amount of proteins produced from a given transcript, whereas the cell contains many other proteins produced in the past that are still present in the cell.

### General features of the yeast gene expression system

**Figure 4:**
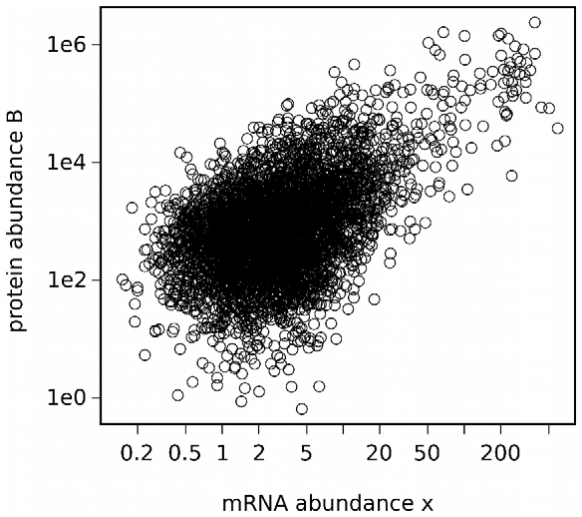
Correlation of mRNA and protein expression levels. The plot shows the correlation between mRNA abundance (parameter 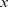) and the number of protein molecules produced from a given gene (parameter 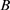). We performed linear regression through the origin on log transformed data. Adjusted 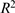 value calculated over the entire dataset (4192 genes of known 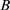) was 0.59. This means that over 40% (in log space) of the variation in protein abundance cannot be explained by variation in mRNA abundance, suggesting some additional, posttranscriptional mechanisms of gene expression regulation.

Based on our results, we can draw the following conclusions concerning gene expression in *S. cerevisiae*:

First, half of the genes produce less than 2.73 transcripts per cell. The distribution of the transcript copy number is skewed with a long right tail: only 55 genes have more than 100 mRNA copies. Unsurprisingly, the top 20 genes with the highest 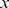 values turned out to be either ribosomal proteins (18 genes) or enzymes engaged in glycolysis (genes YKL060C and YKL152C). One mRNA molecule is translated from 0.14 to 40,110 times, and the median is 257.9. Typically, one gene produces 677 protein copies; however, the most active genes may generate more than 2 million protein copies. Only six genes are common for the sets of the top 20 genes with the highest transcript levels and protein abundance. Among the 20 most highly produced proteins, there are 14 ribosomal proteins, two genes engaged in glycolysis (YCR012W, YKL060C), a highly expressed mitochondrial aminotransferase (YHR208W), alcohol dehydrogenase (YOL086C), and two cell wall proteins (YLR110C, YKL096W-A). There is only partial correlation between transcript and protein copy number and protein production does not necessarily follow the concentration of mRNA molecules (see Figure 4). We compared mRNA (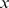) and protein (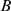) abundance calculated in our model, by performing linear regression through the origin on log transformed data. Adjusted 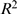 value calculated over the entire dataset (4192 genes with known 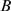) was 0.59. This means that over 40% (in log space) of the variation in protein abundance cannot be explained by variation in mRNA abundance, suggesting some additional, posttranscriptional mechanisms of gene expression regulation.

Next, we analysed yeast genes for expression strategies applied to produce the highest number of protein molecules. We prepared two datasets: 200 genes with the highest 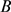 values (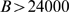) and 200 genes with the lowest 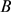 values (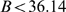). We compared the rest of the translation parameters between these two sets, performing a two-sided Mann-Whitney test. The mean value of most parameters differs between the two datasets in an intuitive manner: genes coding for highly abundant proteins usually produce more transcripts, which have a shorter time of translation (both 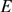 and 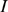), as well as stronger resilience to degradation and are occupied by more ribosomes per 100 codons. All differences are statistically significant with p-value 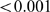 (data not shown). Only one parameter appeared not to affect the number of proteins produced: the relative rate 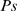 of initiating translation once the ribosome attaches to the free 5′ end of an mRNA molecule (p-value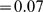). Moreover, the Spearman's correlation coefficient between parameters 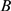 and 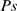 for the entire dataset is very weak (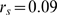, p-value 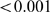).

Analogously, we analysed two datasets of 200 genes with the highest and lowest 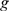 values (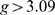 and 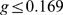, respectively). According to the Mann-Whitney test, transcripts of higher ribosome density typically produce more protein molecules and have shorter times of translation (both 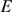 and 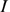). All differences are statistically significant with p-value 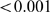 (data not shown). In contrast to the result mentioned above, the shorter time 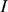 for genes of the highest ribosome density is here caused mainly by elevated 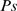, while 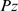 has little influence, but the difference in 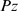 is still statistically significant (p-value 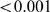). Nevertheless, no significant correlation was observed between the parameters 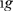 and 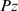 measured over the entire dataset (p-value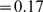). The roles of 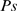 and 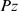 in modifying values of 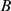 and 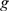 are detailed in Supplementary Figure S2.

Furthermore, we studied, in detail, 20 genes from the set of 200 genes producing the highest number of proteins but with low transcriptional activity (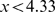 for all of them). Interestingly, these genes are involved in many distinct biological processes, with the notable exception of ribosome formation. The mechanism of their regulation, deduced from the values of the translational parameters, is almost the same for all genes. For instance, two parameters seem to play the main role in sustaining the high protein synthesis rate: relatively long mean life-time of the mRNA molecule, reaching up to several dozens hours (the maximal observed mean lifetime of a yeast transcript is 61 hours), and about four times shorter time of translation initiation, caused mainly by relatively high 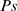 values. On average, the observed 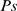 is one order of magnitude higher than the median 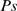 for all yeast genes. The shorter pause between subsequent initiations results in elevated ribosome density 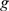 and increased protein production rate. On the other hand, the total time of translation, as well as the mean elongation time, are unexpectedly long (i.e., slightly above the median value of all yeast genes (see Table 3)). This indicated that in cases of long-lived mRNAs, high transcriptional rates and usage of frequent codons are not required to achieve a high rate of protein synthesis. This strategy of expression constitutes an interesting but still inscrutable example of translation regulation, and further research should be carried out.

**Table 3:** Translational parameters of 20 genes of low transcriptional activity and high protein production rate.

parameter	median	min	max
L	753.5	205	1877
x	3.38	1.61	4.30
B	37794	25263	97900
g	3.22	1.35	4.86
w	20.05	8.32	63.25
P	1.7e-4	6.9e-5	3.3e-4
Pz	9.4e-5	4.5e-5	1.2e-4
Ps	2.9e-2	1.0e-2	8.3e-2
T	2:28	0:28	5:31
I	0:06	0:03	0:15
E	2:23	0:25	5:26
mean_E	0.193	0.124	0.213
m	24:09:05	8:18:33	56:26:44

The distribution of translational parameter values for the set of 20 genes having high protein production rates (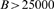) and relatively low transcriptional activity (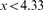). Column descriptions: (1) name of the parameter; (2) median value; (3) minimal observed value; and (4) maximal observed value. The units are the same as those presented in Table 1.

### Translation times and codon bias

In Supplementary Table S2 we present times of translation of individual yeast codons at 30

**Figure 5:**
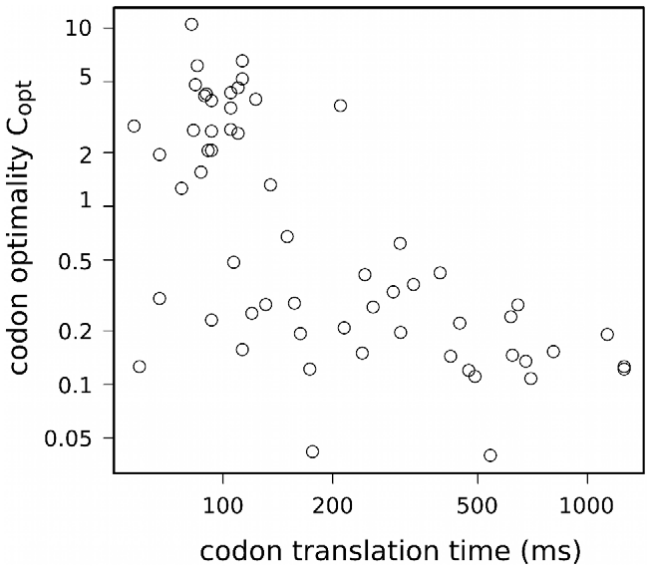
Codon optimality vs translation time. The plot shows the coparison of translation times in 30

C. We compared these values with codon optimality 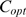 calculated by [36]. The value of 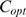 measures whether the codon is preferred in highly expressed genes compared with all other codons encoding the same amino acid. 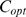 is calculated as the odds ratio of codon usage between highly and lowly expressed genes. Figure 5 shows that there is negative correlation between 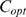 value and translation time of a codon. However, while optimal codons have only short times of translation, non-optimal codons may be translated at both high and low rates. Linear regression model through the origin on log transformed values confirmed this conclusion: the obtained adjusted 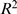 is only 0.15. This indicates, that translation speed may be the one, but not the only criterium for selection on codon bias. This is in agreement with other reports, discussed widely in the recent review [37]. Also, it has been shown that codon usage bias in yeast is associated with translation accuracy [38] and protein structure [36].

C of individual yeast codons with codon optimality values 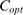 calculated by [36]. There is negative correlation between 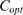 value and translation time of a codon. However, while optimal codons (high 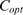 values) have only short times of translation, non-optimal codons may be translated at both high and low rates. Adjusted 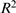 value obtained in linear regression model through the origin on log transformed values indicates, that translation speed may explain only 15% of variability in 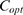 values.

### Translational parameters and protein interactions

Interacting proteins are often precisely co-expressed, presumably to maintain proper stoichiometry among interacting components [39]. For instance, it was shown that functionally associated proteins exhibit correlated mRNA expression profiles over a set of environmental conditions [40], [41]. Other studies report the co-evolution of codon usage of functionally linked genes [39], [42] and show that codon usage is a strong predictor of protein-protein interactions [43]. Our model provides far more information on translation regulation than mRNA expression profiles or codon usage alone, thus we decided to examine calculated parameters in a set of well-known interacting proteins.

As a model, we chose the 20S proteasome complex, built of 28 proteins. There are 14 genes in the yeast genome coding for proteasome subunits 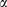
1–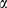
7 and 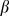
1–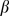
7, and each subunit is present in the complex in two copies [44]. Only subunit 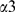 is nonessential for the functionality of the complex and may be replaced by the 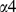 subunit under stress conditions to create a more active proteasomal isoform [45].

The analysis of the translational parameters (see Supplementary, Table S3) shows that the mean translation time (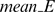) of all proteins is similar and ranges from 194 to 259 ms. As all interacting proteins are of similar length, the total time of elongation does not vary much; the biggest observed difference between two proteins was 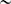
24 s. However, the level of transcription is more variable and ranges from 3.99 to 22.61 transcripts per cell. There is a considerable divergence of ribosome density 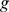 (from 0.61 to 4.32), but regulation at the level of translation initiation (similar values of 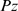 and variability of 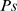 reaching two orders of magnitude) keeps the initiation time 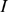 at the same level for all 14 proteins. The biggest observed difference of 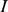 between two proteins equals 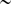
31 s. This results in congruent total times of translation 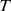, the difference between maximal and minimal values is only two-fold with a mean value of 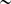
73 s. Nevertheless, the observed differences in the mean lifetimes of mRNA molecules are huge, reaching up to 278 min. In consequence, the number of protein molecules produced is strongly variable, ranging from 318 to 11,185 molecules per cell, and this is surprising as the stoichiometry of the 20S proteasome would rather suggest equal amounts of all subunits. Indeed, for four proteasome proteins, the value of the 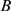 parameter is almost the same, about 2,600 subunits of 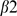, 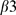, 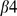, and 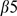 per cell. Similar values, which do not exceed the range 2,600
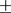
1,000, were reported for subunits 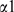, 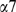, 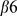, and 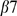. Subunits 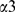, 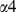, 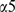, and 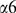 are produced to less than 1,100 copies, while the rate of protein synthesis of subunits 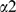 and 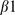 is 5,481 and 11,185 molecules per cell, respectively. To maintain the number of different subunits at the same level, the high translation rates of 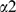 and 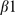 may be balanced by post-translational regulation, presumably by elevated protein degradation. Conversely, the reduced translation rate of 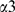, 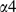, 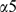, and 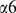 may be compensated at the level of transcription, for instance by more frequent transcription initiations. In addition, the limited number of these subunits, as well as the relatively short life-time of their mRNAs, makes them ideal candidates for regulators of the abundance of proteasome complexes.

## Discussion

The main advantage of the proposed model is that basing it on only few datasets and general assumptions allows the calculation of many important translational parameters, which are extremely difficult to measure experimentally. As a result, the majority of yeast genes may be attributed with quantitative rates of expression and protein synthesis. These data may be used to study both the general characteristics of the process of translation in yeast and the rates of protein production of individual genes. The model itself is general and universal and can be applied to other organisms if all of the necessary input datasets are available.

However, as with any theoretical model, this one also has some drawbacks. The quality of our calculations strongly depends on the quality of the input data. To study the example of *S. cerevisiae*, we carefully chose the dataset of ribosome profiles and made sure that data on mRNA abundance and ribosome footprints were obtained under the same experimental conditions. Similarly, all global parameters, such as the overall number of transcripts and ribosomes in a cell, were determined with care and attention, after insightful analysis of the literature. To extend our model to the number of proteins produced, we decided to use an additional dataset of mRNA half-lives. The assumption that lies at the basis of mRNA half-life calculation is that in the steady state of mRNA turnover, the time required to synthesise an mRNA molecule equals the time to degrade it. Obviously, this is not true for many transcripts, as the cell cycle and environmental stimuli force changes in mRNA turnover. Additionally, we must not forget that the parameter 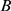, calculated based on mRNA half-life, reflects only the total amount of protein molecules produced by the transcripts of a given gene. The protein degradation rate is not taken into account, and therefore, especially in case of short-lived proteins, the observed protein concentration will be smaller than estimated in this paper. This may be the cause of some of the discrepancies between the estimated protein abundances and those previously reported.

The true meaning of the 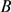 parameter is also important when analysing the set of 20 genes characterised by low levels of transcription and high levels of protein production rate. As their transcripts may be sustained in a cell for up to a few dozen hours, they may produce a large amount of protein in their lifetime, even if the translation is not very efficient. However, this does not necessarily indicate that all synthesised proteins are aggregated in a cell, and their number is constantly increasing. It is more likely that these proteins are systematically degraded and replaced by new ones produced from the same mRNA. Interestingly, genes regulated thusly would not be classified as highly expressed by any standard methods, as their transcripts are not present in the cell in many copies, and their mean time of elongation is about average, so no codon bias is suspected.

In addition, our model revealed some interesting aspects of global translation characteristics. In many studies ribosome density is used as the only measure of translational activity [6]. We have shown that high ribosome density is caused mainly by the elevated relative rate of translation initiation after forming of the ribosome-mRNA complex – 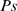. In contrast, another measure of translation efficiency, protein production rate 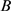, is affected mostly by the relative rate of finding an mRNA molecule by a free ribosome 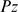, while the influence of 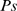 in this case is negligible. These results reflect the complexity of translation regulation, suggesting that any translational parameter, when considered separately, is not sufficient to fully characterise the process.

It has been stated before [46] that the regulation of gene expression is controlled at multiple stages, and no general rule exists describing how it works. In fact, the regulation of expression is different for each gene, and its main role is to produce the required amount of a given protein at the proper time. In contrast to the typically used methods of quantifying translation (i.e., codon bias and transcript abundance measurements), the proposed model does not concentrate only on one parameter of translation. In fact, it allows one to study, in depth, many strategies of gene expression, showing which parameters play the main role in which type of control.

Furthermore, the model opens the prospect for new analysis of mRNA molecules. As mentioned before, the translation initiation rate depends on mRNA abundance and intrinsic features of the transcript. The calculated parameter 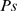 measures the relative efficiency of translation initiation, excluding the influence of mRNA concentration. Thus, for the first time, it provides a quantitative way to compare mRNA sequences from the same organism with respect to initial codon context, 5′UTR secondary structure, 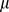
ORF presence, and other mRNA features responsible for the efficiency of translation initiation.

Another possible application of the model is the analysis of the calculated translational parameters in the context of protein complexes, where proper stoichiometry among interacting components is maintained. As exemplified by the study of the yeast 20S proteasome, such analysis enables one to draw some interesting conclusions about the regulation of the individual proteins, as well as the entire complex. Moreover, we have shown that some parameters, in particular translation times 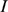, 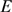 and 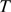, are similar for all proteins of the complex. Possibly, the calculated model parameters, if properly integrated, could become a strong predictor of protein-protein interactions. It would be interesting to carry out a similar search for proteins participating in the same metabolic pathway, as functionally related proteins are usually co-expressed. In such a case, the analysis of the translational parameters pattern could become useful for the functional annotation of genes.

The model can also be used to study the elongation process in the context of ribosome queuing. It provides all the necessary tools to deeply analyse the strategies developed by living cells to avoid ribosome stacking on a translated mRNA molecule.

Additionally, clustered codons that pair to low-abundance tRNA isoacceptors cause local slow-down of the elongation rate. It has been hypothesised, that such slow-down might facilitate the co-translational folding of defined protein segments, by temporally separating their synthesis [47]. Recently, it has been proposed that discontinuous elongation of the peptide chain can control the efficiency and accuracy of the translation process [48]. Our model provides the measure of yeast codon elongation rates that may be used to better examine the co-translational folding. In contrast to the measure used in the aforementioned study, it is quantitative and more precise, as it takes into account the delay caused by near- and non-cognate aa-tRNAs.

Finally, the crucial coefficients of the model, i.e., the time of insertion of cognate aa-tRNAs and time delays caused by near- and non-cognate aa-tRNAs binding, can be calculated with respect to different temperatures. This provides the possibility to study the excess to which the temperature affects the efficiency of translation, provided that the ribosome footprints and mRNA concentrations are also measured at a few different temperatures.

In conclusion, although experimental confirmation is still required, this model constitutes an important tool for understanding the process of protein synthesis.

## Materials and Methods

### Theoretical model of translation

The molecular mechanism of translation was well characterised previously [49]. However, for the purpose of this research, we must consider the process both at the single transcript and genome-wide levels. Quantifying the process of protein biosynthesis engages vast array of data, some of which is incomplete or missing. Thus, the following assumptions and simplifications must be made: (i) the pools of all molecules participating in translation (mRNA, tRNA, ribosomes, translation factors, and so on) are constant, and molecules diffuse without restraint; (ii) all transcripts derived from the same gene have identical sequence, i.e., there is no alternative splicing and/or posttranscriptional modification; and (iii) the elongation process is never interrupted, and it always ends by producing a full-length protein molecule (note, that experimentally estimated procesivity of translation in yeast was 99.8–99.9% [50]). When these assumptions are satisfied, the model is as follows:

Let 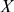 be the set of all transcripts present in the yeast cell at the moment of observation. We can make a partition of the set 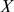 into 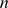 subsets, each containing transcripts of identical sequence. Thus, 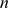 denotes the number of transcriptionally active genes in the cell. To each gene (subset), we attribute the index 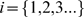 and define 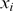 as the number of transcripts in the 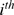 subset. The variable 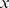 is reflected though by the transcriptional activity of a gene.

Let 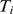 be the total observed time of synthesis of one protein molecule from a transcript belonging to the 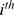 subset. We define it as:
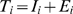
(1)where 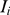 denotes the time required for translation initiation, and 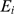 is the total time of the elongation process.

We define 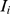 as the time interval from the point when the free 5′ end of a transcript becomes available for ribosomes to the moment when a ribosome finds the initiator AUG codon and the entire complex enters into the elongation phase. The inverse of the initiation time 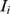 is initiation frequency 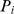:
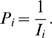
(2)If these frequencies are multiplied by a brief time interval 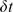, one obtains the probabilities that the initiation process will occur during time interval 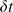. We assume that the initiation of translation follows the scanning model [51], which postulates that the small ribosomal subunit enters at the 5′ end of the mRNA and moves linearly, searching for the initiator AUG codon; once it finds it, the elongation process begins. We define 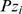 as the relative binding rate of free ribosomes to the 5′ end of the 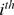 transcript, and assume it is proportional to the concentration of the transcript (see Eq.10). This means, that in our model the binding constants of ribosomes are the same for all mRNAs. Contrary, the process of 5′UTR scanning by the ribosome is not straightforward, as there are many intrinsic features of mRNA molecules that can considerably delay or hasten the start of elongation (for review, see [1]). Sometimes, the ribosome detaches from the mRNA molecule before reaching the initial AUG, and the process must return to the point when a ribosome binds at the 5′ end. To describe the efficiency of the scanning process by one numerical parameter, we normalised 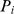 by the rate of binding of free ribosomes 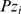:
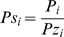
(3)The calculated parameter 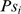 describes the rate of successful accomplishment of initiation on the 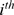 transcript once the ribosome-mRNA complex is formed. Its value reflects the relative capability of an mRNA molecule to be translated, regardless its expression level. The rates 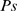 and 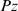 are calculated in relation to all studied transcripts, thus they can only be compared within one particular analysis.

The time 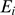 (see Eq.1) is defined as a time interval from the recognition of the initiator AUG codon by the ribosome to the moment when the last peptide bond of a protein molecule is formed. Each elongation event consists of two main steps: (i) finding the correct tRNA molecule, and (ii) formation of the peptide bond and translocation. The time required for the first event is much larger than for the second. In fact, the second step is almost instantaneous [52]; thus, the times needed for transpeptidase and translocation reactions can be neglected, and time 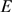 may be simplified to:
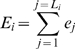
(4)where 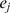 is the time of translation of the 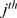 codon, and 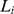 is the number of codons in the coding sequence of the 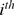 transcript.

Translation times for all yeast codons, as well as the values of 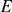 and 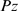, can be calculated on the basis of existing data (see below). These values can also be used to calculate times 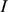 and the rest of the model parameters 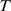, 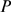, and 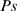, if the numbers of ribosomes attached to the mRNA molecules are known. Here, the reasoning is as follows:

Let 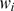 be the number of ribosomes attached to the 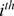 transcript. We introduce the measure of ribosome density 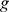, defined as the number of ribosomes attached to the transcript per 100 codons:
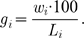
(5)One ribosome occupies ten codons of a mRNA molecule [53], and the E site of one ribosome can be immediately adjacent to the A site of another ribosome [54]. This means that the maximum possible value is 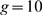. Next, the attachment of a ribosome to the 5′ end is possible only if it is not occupied by other ribosomes. Thus, the most efficient mRNA sequences should have 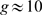. Nevertheless, the majority of observed 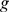 values are much smaller, meaning that there are usually gaps of varying length between attached ribosomes. As the exact positions of ribosomes on a particular transcript cannot be deduced from the data, we must operate on the averaged gap lengths, defined as the quotient of the transcript length 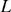 and number of attached ribosomes 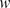. The length of a gap measured in codons is meaningless, as each type of codon has a different translation time. However, the gap can be calculated as the sum of translation times of these codons, becoming an adequate measure of the time interval between individual translation initiation events on a given mRNA molecule. This time is actually a delay from the best possible initiation frequency and reflects the efficiency of the initiation process. In principle, this is the time 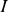 (see Eq. 1) expressed in the same time units as the translation times of particular codons:
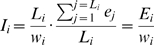
(6)Note that due to unknown ribosome positions on a transcript, both the gap length and time of its translation are averaged. The rest of the parameters (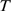, 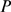, and 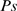) can be calculated based on 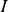, as shown in Eq. 1, 2, and 3.

### Calculating model parameters

The *S. cerevisiae* coding sequences used in our calculations were downloaded from the Saccharomyces Genome Database [55] (accessed 25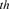 June 2009). For each gene, we determined the values of 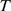, 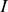, 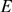, 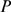, 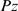, and 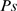 on the basis of the recent research of Ingolia et al. [22], quantifying simultaneously mRNA abundance and ribosome footprints by means of deep sequencing. The study was done for the yeast strain BY4741 grown in YEPD at 30

C. In the first step Ingolia et al. performed deep sequencing on a DNA library that was generated from fragmented total mRNA in order to measure abundance of different yeast transcripts. Next, they applied a new ribosome-profiling strategy based on the deep sequencing of ribosome-protected fragments. This resulted in a dataset of 4,648 reliable transcripts (for the definition of “reliability”, see Supplementary Materials of [22]) that was used as an input in our research. For each transcript in the dataset, the following values were attributed: 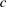, raw count of mRNA-seq reads aligned to transcript coding sequence (CDS); 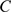, density of mRNA-seq reads in reads per kilobase per million CDS-aligned reads (RPKM); 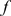, raw count of ribosome CDS-aligned footprints; and 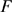, density of ribosome footprints in reads per kilobase per million CDS-aligned reads. Next, the relative numbers of reads counted in RPKM were transformed into the transcript copy numbers. Normally, for each transcript 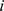, 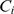 is defined as:
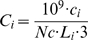
(7)where 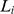 is the length of the transcript CDS in codons, and 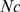 is the sum of all mappable reads 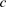 [56]. Assuming uniform distribution of the mappable reads across the transcriptome coding sequences, the probability of observing 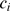 reads on the 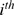 transcript CDS of length 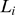 in 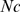 attempts corresponds to the fraction of the transcriptome composed of the 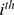 transcript:
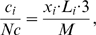
(8)where 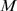 is the sum of all CDS of the transcriptome in base pairs. The meaning of 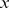 was explained in the previous section. We can substitute final RPKMs to get:
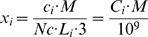
(9)


Although the length of the entire transcriptome was estimated as 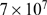 nucleotides [57], deriving 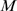 is more problematic, as little is known about the accurate boundaries of non-coding elements in transcript sequences [9]. There were some attempts to determine the length of UTRs on a global scale in yeast [58], [59], but the results show that even the length of transcripts derived from the same gene of the same yeast strain cultured in the same growing conditions may vary considerably. This causes the discrepancies between reported transcript lengths by these two studies, making the analysis at the level of individual genes difficult and inaccurate.

To overcome this problem we use 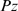, the relative rate of binding of free ribosomes to the 5′end of a given transcript (see Eq.3). This rate corresponds to the fraction of transcript 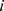 in the set of all transcripts. By substituting 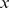 as shown in Eq. 9, we obtained the following relation:
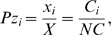
(10)where 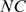 is the sum of all densities 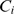 of mRNA-seq reads. Thus:
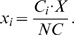
(11)


The next step was to calculate 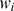 (the absolute number of translationally active ribosomes attached to the 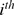 transcript), and 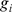 (the measure of ribosome density, as defined in Eq.5). The dataset used provides information only on ribosome footprints aligned to the coding sequences. However, in practice, there were some exceptions to this rule, caused mostly by the presence of 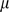
ORFs in the 5′UTR sequences [22]. Due to the lack of data and aforementioned difficulties in determining exact transcript length, this fact is not taken into account in our analysis. Furthermore, we defined 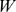 as the number of all ribosomes in a yeast cell and 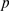 as the fraction of ribosomes participating in the process of translation at the moment of observation. In contrast to raw mRNA-seq reads, the distribution of ribosome footprints is not uniform across the transcriptome, due to differences in genes translational activity. Thus, the probability of observing a ribosome attached to the 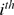 transcript corresponds to the fraction of all ribosome footprints 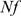 composed of the raw footprints in the 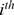 transcript, 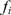. This probability is equal to the ratio of all ribosomes engaged in translation of transcripts of type 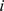 and the number of all occupied ribosomes in the cell:
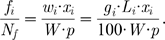
(12)Thus, ribosome density for the 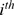 transcript can be calculated as:
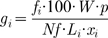
(13)


### Global parameters estimation

Three parameters must be estimated to transform relative numbers of transcripts and ribosomes attached to them into absolute measures. These parameters are 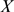, the total number of mRNA transcripts in a yeast cell; 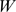, the total number of ribosomes in a yeast cell; and 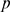, the fraction of ribosomes participating in the translation process at the moment of observation. There are many studies concerning the quantitative measurement of yeast cells, and we used the Bionumbers database [60] to extract these data.

Two reports provide an independent, yet coherent, estimation of the total number of ribosomes: 187,000
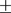
56,000 [9] and 200,000 [57] molecules per cell. In this study, we decided to set 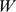 to 200,000. The value of 85% was established for the parameter 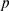, as stated in experimental studies [61], [62]. The number of all transcripts in a cell is more problematic. Many contemporary studies assume that a yeast cell contains 15,000 mRNAs per cell on average [27], [63], which is based on estimations done over 30 years ago [64]. Current research, based on more up-to-date techniques (e.g., *in situ* hybridisation or GATC-PCR) argues that the number should be at least doubled [65] or even quadrupled [62]. We decided to use the value of 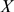 situated between these estimates and equal to 36,000. This number was also confirmed by other studies [65].

Assuming 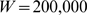, 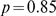, and 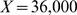, we obtained the mean ribosome density equal to 1.66 ribosomes per 100 codons. This is in agreement with experimental analysis, which reports that, on average, there is one ribosome per 156 nucleotides, corresponding to a density of 1.92 ribosomes per 100 codons [61]. Moreover, it was estimated that mRNA constitutes 5% of the total amount of RNA present in a cell, and the RNA∶DNA ratio is 50∶1 [57]. Assuming the yeast genome size of 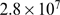 nucleotides, the expected length of the entire transcriptome would be 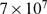 nucleotides. Thus, the length of all transcribed coding sequences 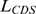 can be defined as:
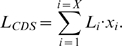
(14)The meanings of 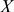, 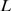, and 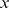 are explained above. Thus, the calculated length of all coding sequences equals 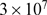 nucleotides. This would suggest that non-coding elements constitute, on average, more than 50% of a transcript. In conclusion, it seems that the chosen parameter values generate reasonable measures of the global characteristics of the yeast cell.

### Determining absolute times of translation

In the previous section, we calculated the values of 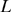, 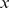, 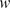, 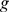, and 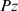 for each gene. To determine the absolute times of translation 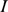, 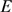, and 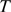, we need to know the times of translation for individual codons. To achieve this goal, we adapted a model proposed for *Escherichia coli* [66] to the yeast system. Here, we briefly present the model and all of the necessary changes we made. For a description of the derivation, see the original paper.

The transport mechanism in the cytoplasm is diffusion, thus the aa-tRNAs act as a random walker, and the ribosomes on mRNAs with vacant A sites are the targets. We assume a yeast cytoplasm volume 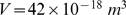 [67]. We divide it into 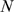 walker occupation sites, where:
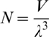
(15)and 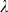 is a measure of the walker size. The values of 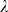 used previously [66] were determined separately for individual *E. coli* aa-tRNA molecules [68]. As we are not aware of any similar reports for *S. cerevisiae*, we decided to use 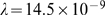 for all yeast codons, which is the mean of the *E. coli* 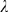 values. The average time that elapses before the arrival of a walker 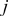 is defined as:
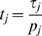
(16)where 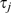 is the characteristic time of the 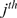 walker, associated with its transition from one cellular occupation site to the other. It depends on the size of the walker 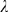 and its diffusion coefficient 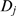:
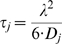
(17)The measures of 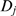 were taken directly from [66]. As this value depends only on the accepted amino acid, we assumed that the difference in size between yeast and *E.coli* tRNA molecules is negligible. In Eq.16, 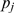 stands for the probability that a tRNA-aa molecule of type 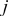 arrives at an open A site in the time interval 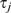 and is proportional to the number of walker occupation sites containing the 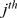 walker:
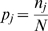
(18)We assume that the number of the molecules of the 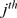 walker 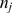 is proportional to the number of corresponding tRNA genes of type 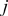, which is reasonable, as it was shown that in yeast the concentration of the various tRNA species is largely determined by tRNA gene copy number [69]. In particular, the calculated correlation coefficient between tRNA gene copy number and experimentally determined tRNA abundance for a subset of 21 tRNA species equaled 0.91. According to [57], the RNA-DNA ratio is 50∶1 and tRNA constitutes 15% of the total amount of RNA in a yeast cell. Assuming a genome size of 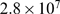 nucleotides, the total cellular tRNA size is 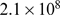 nucleotides. When divided by the average tRNA molecule size (74.5 nt) we obtain the number of tRNA molecules in a cell equal to 2,818,792. Next, this number was multiplied by the fraction of all tRNA genes composed of the tRNA genes of type 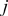, yielding the absolute amount of particular tRNA molecules in a cell. Gene copy number and predicted decoding specificities of yeast tRNAs were taken from Table 1 of [69]. The values of all presented parameters for individual tRNAs are gathered in Supplementary Table S4.

All 61 codons that code for the 20 amino acids have one or more aa-tRNAs and varying numbers of near-cognates. Near-cognates are defined as having a single mismatch in the codon-anticodon loop in either the 2nd or 3rd position. Since some cognate tRNAs have a mismatch in the 3rd position, these tRNAs are excluded from the set of near-cognates [70]. The theoretical background of the model is based on the observation that the translation rate of a codon reflects the competition between its non-cognate, near-cognate and cognate aa-tRNAs [71], and that such nonspecific binding of the tRNAs to the ribosomal A site is rate-limiting to the elongation cycle for every codon [72]. The model of Fluitt et al [66] introduces two competition measures, 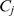 and 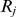, being the quotients of the sum of arrival frequencies of near-cognates vs. cognates and non-cognates vs. cognates, respectively. For each codon, we determined its cognates, near-, and non-cognates (based on [69]) and calculated the competition measures 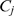 and 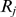 (see Supplementary Table S2).

According to [66], the average time to add an amino acid coded by the 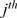 codon to the nascent peptide chain can be calculated as:
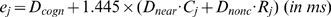
(19)where 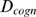 is the average time to insert an amino acid from a cognate aa-tRNA, and 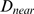 and 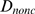 are the average time delays caused by the binding attempts of near- and non-cognate tRNAs, respectively. Based on existing data and assumption that the activation energies for the various reactions do not vary much, Fluitt et al [66] showed how to calculate the values of 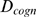, 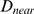 and 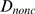 at any given temperature. Table 4 contains these values for *S. cerevisiae* at 20, 24, 30, and 37

C. Next, we calculated translation rates 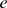 for all yeast codons at the four different temperatures (see Supplementary Table S2). However, as the main part of our analysis is based on the ribosome footprints measured at 30

C, in further calculations we use only the values of 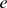 estimated at this temperature. The last step was to calculate times 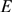 for individual 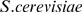 genes, as described in Eq.4.

**Table 4:** Time of tRNAs insertions at four different temperatures.

temp	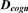	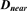	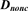
20  C	40.0	46.3	02.2
24  C	26.6	30.7	01.5
30  C	16.1	18.7	00.9
37  C	09.1	10.5	00.5

Values of 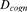, 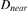, and 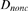 coefficients at four different temperatures. 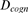 is the average time to insert an amino acid from a cognate aa-tRNA, 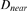 and 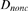 are the average time delays caused by the binding attempts by near- and non-cognate tRNA, respectively. All times are in ms.

### Ribosome queuing

It has been found that subsequent ribosomes are loaded onto the transcript sufficiently fast to make them interfere with each other, leading to ribosome queuing [73]. This phenomenon is usually caused by the presence of rare codons clusters in CDS, although other sequence features may also be very important [74]. Such elongation pauses may have distinct consequences, for instance ORF shifting or ribosome dissociation, often followed by decay of the mRNA and partly completed protein products [75]. Moreover, stalled ribosomes generate a false picture of a transcript translational activity, elevating the observed ribosome density in relation to the actual frequency of translation initiation events. For these reasons, we decided to reduce the dataset to the transcripts on which ribosome queuing does not occur. We wrote a simple program that simulates the ribosomes translocation along a transcript sequence. A ribosome moves from one codon to another only if it has spent a required amount of time for translation of the current codon (taken from Supplementary Table S2) and the subsequent codon is vacant. The successive ribosome attempts to attach to the initial AUG codon after the elapse of time interval 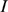, calculated as shown in Eq.6. The cumulative time of the movement is calculated for each ribosome separately. If this time is identical for each ribosome, translation is believed to pass without ribosome queuing. If the time is different, namely, the first ribosome moves faster than the rest, it means that some sequence features allow ribosome stacking under the assumed conditions (i.e., temperature and translational parameters). If the attachment of subsequent ribosomes is prevented by very slow translation of the first few codons, we consider it a particular case of ribosome queuing and reject all such transcripts.

### Calculation of protein abundances

To enrich our dataset, we estimated the total number of proteins produced from a given transcript. Considering the mRNA molecules as a decaying quantity, we defined 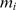 as the mean lifetime of the 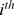 transcript expressed in time units:
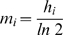
(20)where 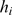 is the half-life of the 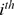 transcript. Assuming that each translation event happens independently, we calculated the abundance of the 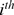 protein as the number of translation initiation events that happen during the life-time of the 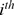 transcript multiplied by the its copy number:
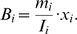
(21)The dataset of mRNA relative half-lives is provided in the Supplementary Materials of [25]. In our calculations, we used the times *t0* measured at exponential growth in YPD medium for 5,718 ORFs. It was determined experimentally by independent studies that the absolute mRNA half-life of the yeast gene YOR202W (HIS3) ranges from 7 (at 24

C) [76] to 11 min (at 30

C) [77]. Assuming the mean value of 9 min for this gene, we can quantify the half-lives for the rest of the genes in the dataset, as well as the values of 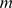 and 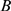.

### Calculations summary

Based on the presented reasoning, we calculated translational parameters for the majority of yeast genes. In particular, parameter 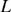 was calculated on the basis of yeast coding sequences downloaded from [55]. Parameter 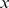 was obtained from Eq.11, where values of 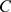 and 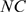 were taken from the experimental study [22], and 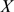 was set to 36,000, as estimated by [65]. Parameter 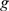 was obtained from Eq.13, where values of 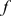, 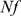 were taken from [22], 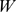 was set to 200,000, based on [57], and 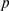 was set to 0.85 as stated in [61], [62]. Parameter 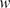 was calculated from Eq.5. Parameter 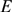 was calculated from Eq.4, based on yeast coding sequences downloaded from [55] and the values of translation times of codons 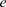, calculated as shown in Eq.19. The values of 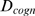, 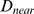 and 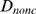 (at 30

C) used in Eq.19 were calculated as shown in [66], and the values of 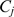 and 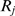 were calculated separately for each codon as shown in [66], by substituing the number of its cognates, near-, and non-cognates tRNAs determined on the basis of [69]. Parameter 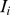 was obtained from Eq.6, and 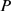 from Eq.2. Parameter 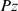 was obtained from Eq.10, where values of 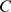 and 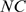 were taken from the experimental study [22]. Parameter 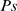 was obtained from Eq.3 and then normalised by its maximal value reported for the gene YLL040C. Total time of translation 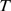 was calculated as stated in Eq.1. Mean time required for elongation of one codon of the 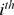 transcript (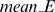) was calculated by dividing elongation time 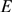 by the length of this transcript in codons 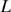. Parameter 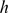 was obtained on the basis of relative half-lives for yeast transcripts reported by [25] and mRNA half-life of the yeast gene YOR202W, assumed to be on average 9 min [76], [77]. Parameter 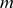 was obtained from Eq.20, and 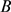 from Eq.21. The meaning of all variables was presented at the beginning of this section.

## Supporting Information

Figure S1 The comparison of model parameters x and B with experimentally determined mRNA and protein abundances.
(0.26 MB PDF)

Figure S2 The comparison of translational parameters between genes of high and low protein abundance, as well as between genes of high and low ribosome density.
(0.23 MB PDF)

Table S1 A separate csv file containing calculated quantitative measures of translation for 4,621 yeast genes. There are 151 genes for which ribosome queuing was reported (parameter queue ! = 0, see below); the values of translational parameters of these genes may be irrelevant. Column descriptions: (gene) the systematic name of the yeast gene taken from Saccharomyces Genome Database; (L) length of the transcript CDS in codons; (x) absolute number of gene transcripts in a yeast cell; (b) absolute number of proteins produced from one molecule of a transcript during its lifespan; (B) total amount of protein molecules produced from transcripts of a particular type (B = b * x); (g) ribosome density in number of ribosomes attached to a transcript per 100 codons (g< = 10); (w) absolute number of ribosomes attached to one transcript; (P) translation initiation frequency (the inverse of I); (Pz) relative rate of binding of free ribosomes to the 5′ end of a transcript; (Ps) relative rate of successful accomplishment of initiation once the ribosome-mRNA complex is formed; for clarity, normalised by the maximal observed value of Ps (65.88365), reported for gene YLL040C; (T) total time of translation of one protein molecule from a given transcript in ms (T = I + E); (I) total time (in ms) required for translation initiation, defined as a temporal interval from the point when the free 5′ end of a transcript becomes available for ribosomes to the moment when a ribosome finds the initiation AUG codon and the entire complex starts the phase of elongation; (E) total time required for translation elongation of a transcript in ms; (mean_E) mean time required for elongation of one codon of a transcript in ms; (h) estimated half-life of a transcript in ms; (m) estimated mean lifetime of a transcript in ms; and (queue) ribosome queuing index estimated at 30 Celcius degree: value “0” - no ribosome queuing was observed for a transcript, value “1” - ribosome queuing was observed for a transcript, and value “2” the translation at the 5′ end of a transcript is slow enough to delay the attachment of the successive ribosomes to the mRNA molecule.
(0.58 MB CSV)

Table S2 The list of codons and their properties.
(0.02 MB PDF)

Table S3 The translational parameters calculated for 14 genes coding proteins of the 20S yeast proteasome.
(0.02 MB PDF)

Table S4 Decoding specificities of yeast tRNAs and calculated values of the model parameters for particular codons.
(0.02 MB PDF)
