# Supplementary material for: A Comprehensive, Quantitative, and Genome-Wide Model of Translation
Source: PLoS Comput Biol. 2010 Jul 29;6(7):e1000865. doi: 10.1371/journal.pcbi.1000865 (PMC2912337; doi:10.1371/journal.pcbi.1000865)

Figure S1: The comparison of model parameters x and B with experimentally determined mRNA and protein abundances [13, 26]. The plots show the distribution of the log-fold differences of the mRNA (left) and protein (right) concentrations reported by the model and reference study. Our calculations slightly overestimate the transcript copy number and underestimate the protein copy number in relation to published data. The shift is probably caused by the different total number of transcripts per cell assumed in our model and the fact that the parameter B reflects only the proteins produced by a given transcript and not already present in a cell.

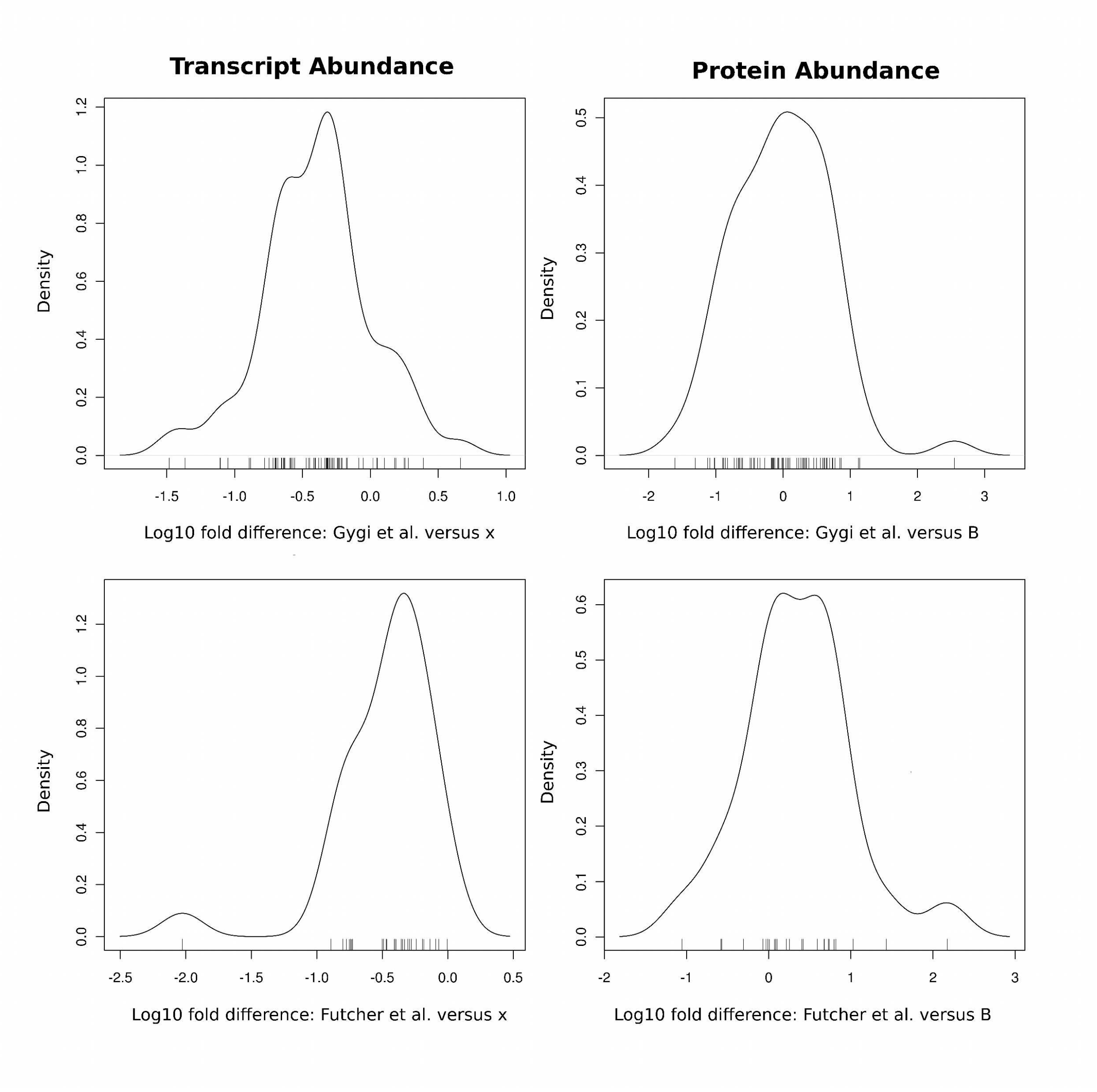

Supplement: Figure S1 — The comparison of model parameters x and B with experimentally determined mRNA and protein abundances. (0.26 MB PDF) [file pcbi.1000865.s001.pdf]
