# Supplementary material for: A Comprehensive, Quantitative, and Genome-Wide Model of Translation
Source: PLoS Comput Biol. 2010 Jul 29;6(7):e1000865. doi: 10.1371/journal.pcbi.1000865 (PMC2912337; doi:10.1371/journal.pcbi.1000865)

Figure S2: From the main dataset, four subsets were extracted, each containing 200 records: genes of the lowest B (number of protein molecules produced), genes of the highest B, genes of the lowest g (ribosome density), and genes of the highest g. We compared the translational parameters between genes of high and low B, as well as between genes of high and low g. Genes having elevated values of B or g tend to have shorter times of translation initiation. In cases of genes with elevated B, the difference is caused mainly by the elevated value of parameter Pz (the relative rate of binding of free ribosomes to the 5' end of a transcript), while the parameter Ps (the relative rate of successful accomplishments of initiation once the ribosome-mRNA complex is formed) seems not to affect the time of translation initiation significantly. On the other hand, genes with elevated g have shorter times of initiation, because of the elevated value of the Ps parameter, while the Pz parameter seems not to affect the time of translation initiation significantly. The boxplots show the distributions of the values of Ps and Pz in all four sets of genes: (A) comparison of Pz values between genes of high and low protein production rate B; (B) comparison of Ps values between genes of high and low protein production rate B; (C) comparison of Pz values between genes of high and low ribosome density g; and (D) comparison of Pz values between genes of high and low ribosome density g.

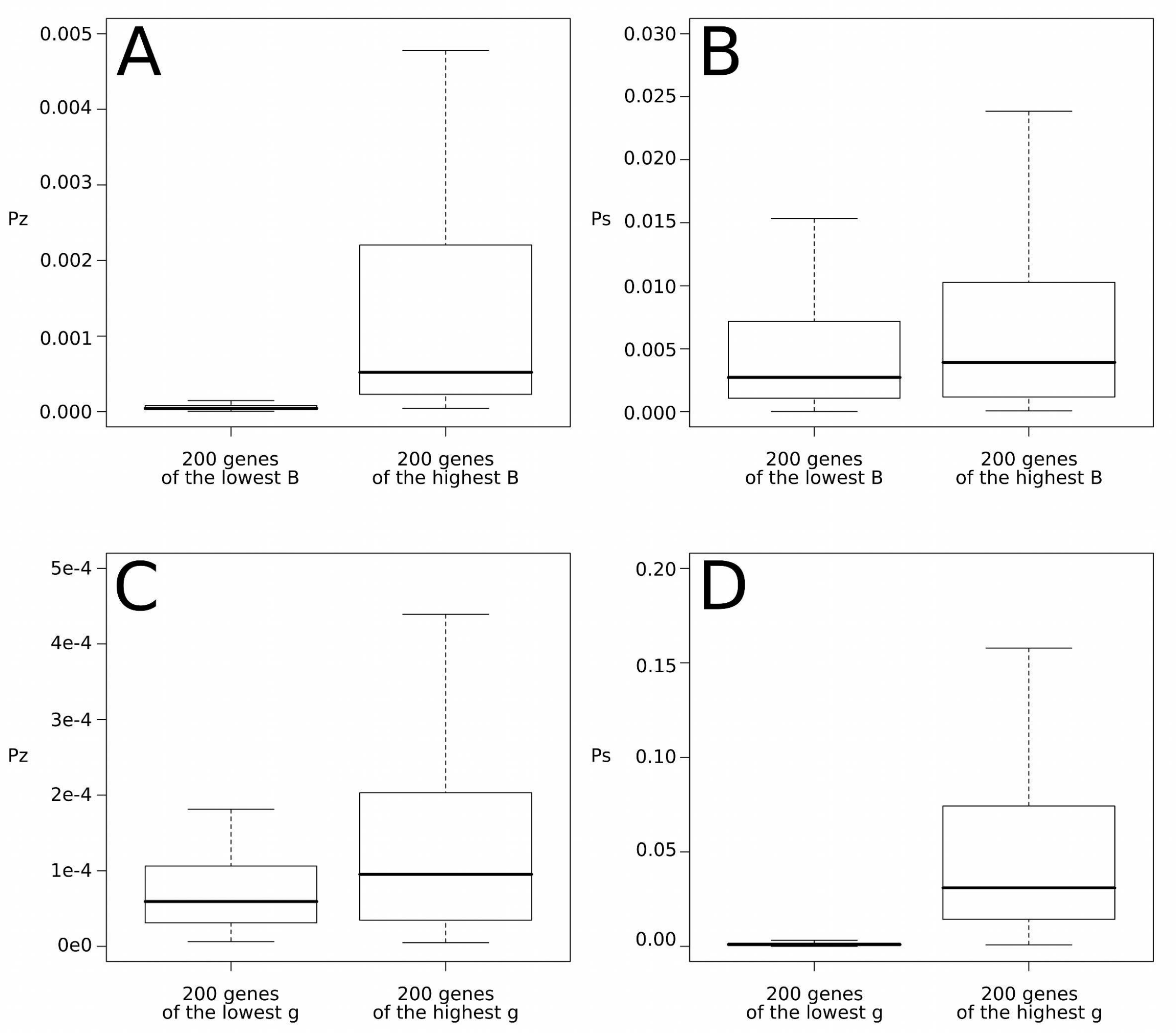

Supplement: Figure S2 — The comparison of translational parameters between genes of high and low protein abundance, as well as between genes of high and low ribosome density. (0.23 MB PDF) [file pcbi.1000865.s002.pdf]
