# Supplementary material for: A Comprehensive, Quantitative, and Genome-Wide Model of Translation
Source: PLoS Comput Biol. 2010 Jul 29;6(7):e1000865. doi: 10.1371/journal.pcbi.1000865 (PMC2912337; doi:10.1371/journal.pcbi.1000865)
Supplement: Table S2 — The list of codons and their properties. (0.02 MB PDF) [file pcbi.1000865.s004.pdf]

Table S2: The list of codons and their properties. Column descriptions: (1) codons recognised by a tRNA molecule; (2,3) cognate and near-cognate tRNAs labelled as per Table S4; (4) competition rate C; (5) competition rate R; and (6,7,8,9) translation times at 20, 24, 30, and 37°C, respectively. All times of translation are in ms.

codon	cognate_tRNAs	near-cognate_tRNAs	C	R	e_{20}	e_{24}	e_{30}	e_{37}
AAA	24	3,7,18,25,34	5.82	31.51	531	353	215	120
AAC	7	24,25,30	2.47	23.07	279	185	113	63
AAG	25	4,7,24,26,35	1.72	16.45	208	138	84	47
AAU	7	19,24,25,36	4.42	21.12	404	268	163	92
ACA	34	3,18,24,35,36	7.94	58.89	761	505	307	172
ACC	36	7,30,34,35	1.78	22.29	231	153	93	52
ACG	35	4,25,26,34,36	34.77	234.07	3121	2072	1260	707
ACU	36	19,34,35	1.68	22.39	224	149	91	51
AGA	3	4,18,24,30,34	1.67	22.39	224	148	90	51
AGC	30	3,4,7	5.51	61.31	605	402	245	137
AGG	4	3,25,26,30,35	34.77	234.07	3121	2072	1260	707
AGU	30	3,4,19,36	8.95	57.88	825	548	333	187
AUA	18	3,19,24,26,34	19.79	113.39	1730	1149	699	392
AUC	19	7,18,26,30	1.61	18.03	206	136	83	47
AUG	26	4,18,19,25,35	6.30	47.26	614	407	248	139
AUU	19	18,26,36	1.36	18.29	190	126	77	43
CAA	10	11,17,20,28	2.32	26.17	279	185	113	63
CAC	17	10,11,21	1.58	35.75	260	173	105	59
CAG	11	5,10,17	17.19	251.65	1997	1326	806	452
CAU	17	6,10,11,29	2.57	34.77	323	215	131	73
CCA	28	10,20,29	1.39	24.15	210	140	85	48
CCC	29	17,21,28	9.05	124.13	1044	693	421	236
CCG	28	5,11,29	0.39	25.15	146	97	59	33
CCU	29	6,28	7.99	125.19	976	648	394	221
CGA	6	5,10,20,28	3.95	40.76	435	289	176	99
CGC	6	5,17,21	1.53	43.18	280	186	113	64
CGG	5	6,11	6.90	261.93	1339	889	541	303
CGU	6	5,29	0.51	44.20	215	143	87	49
CUA	20	10,21,28	6.77	82.17	757	502	306	171
CUC	21	17,20	10.04	258.80	1540	1022	622	349
CUG	20	5,11,21	1.00	87.95	388	257	157	88
CUU	21	6,20,29	10.91	257.92	1595	1059	644	361
GAA	12	1,8,13,14,39	1.93	16.24	221	147	89	50
GAC	8	12,13,16	2.15	14.55	231	153	93	52
GAG	13	8,12,15,40	16.58	116.60	1525	1012	616	345
GAU	8	2,12,13,41	2.68	14.01	265	176	107	60
GCA	1	2,12,14,39	6.04	47.52	597	396	241	135
GCC	2	1,8,16	3.42	20.65	335	223	135	76
GCG	1	2,13,15,40	3.40	50.16	428	284	173	97
GCU	2	1,41	1.77	22.30	230	153	93	52
GGA	14	1,12,15,16,39	13.23	75.72	1170	777	472	265
GGC	16	8,14,15	1.22	14.00	167	111	67	38
GGG	15	13,14,16,40	11.76	121.42	1217	808	491	276
GGU	16	2,14,15,41	1.79	13.43	203	135	82	46
GUA	39	1,12,14,40,41	18.95	114.23	1677	1113	677	380
GUC	41	8,16,39,40	2.55	15.62	261	173	105	59
GUG	40	13,15,39,41	10.00	123.18	1104	733	446	250
GUU	41	2,39,40	1.05	17.12	165	110	67	37
UAC	38	9,27	1.78	31.14	259	172	105	59
UAU	38	33	1.35	31.56	231	154	93	52
UCA	31	22,32,33	6.27	82.68	725	481	293	164
UCC	33	9,27,31,32,38	2.42	21.64	271	180	110	61
UCG	32	23,31,33,37	29.83	239.00	2805	1862	1133	635
UCU	33	31,32	0.37	23.70	140	93	57	32
UGC	9	27,37,38	6.04	60.79	639	424	258	145
UGG	37	9,23,32	2.57	42.14	347	230	140	79
UGU	9	33,37	4.19	62.64	521	346	210	118
UUA	22	23,27,31	3.32	34.02	371	247	150	84
UUC	27	9,22,23,38	2.87	22.67	305	202	123	69
UUG	23	22,27,32,37	2.37	23.17	273	181	110	62
UUU	27	22,23,33	2.75	22.79	297	197	120	67
