# Supplementary material for: A Comprehensive, Quantitative, and Genome-Wide Model of Translation
Source: PLoS Comput Biol. 2010 Jul 29;6(7):e1000865. doi: 10.1371/journal.pcbi.1000865 (PMC2912337; doi:10.1371/journal.pcbi.1000865)
Supplement: Table S3 — The translational parameters calculated for 14 genes coding proteins of the 20S yeast proteasome. (0.02 MB PDF) [file pcbi.1000865.s005.pdf]

Table S3: The translational parameters calculated for 14 genes coding proteins of the 20S yeast proteasome. Row descriptions: (gene) systematic name of the gene; (subunit) name of the corresponding proteasomal subunit; (L) length of the coding sequence in codons; (x) number of transcripts per cell; (B) number of synthesised protein molecules per cell; (g) ribosome density in ribosomes per 100 codons; (w) absolute number of ribosomes per transcript; (P) translation initiation frequency (the inverse of I); (Pz) the relative rate of binding of free ribosomes to the 5' end of a transcript; (Ps) the relative, normalised rate of a successful accomplishment of translation initiation once the ribosome-mRNA complex is formed; (T) total time of translation of one protein molecule from a given transcript in clock notation (min:sec); (I) total time required for translation initiation in clock notation (min:sec); (E) total time required for translation elongation of a transcript in clock notation (min:sec); (mean_E) mean time required for elongation of one codon of a transcript in sec; (h) estimated half-life of a transcript in clock notation (h:min:sec); and (m) estimated mean life-time of a transcript in clock notation (h:min:sec).

gene	YGL011C	YML092C	YGR135W	YOL038W	YGR253C	YMR314W	YOR362C
subunit	alpha-1	alpha-2	alpha-3	alpha-4	alpha-5	alpha-6	alpha-7
L	253	251	259	255	261	235	289
x	5.12	8.95	20.43	16.19	16.20	14.03	13.06
B	3488	5481	972	624	318	1038	3076
g	2.77	1.64	0.77	0.76	1.40	1.03	0.8
w	7.01	4.11	1.99	1.94	3.67	2.42	2.34
P	1.3e-04	7.2e-05	3.7e-05	2.9e-05	6.4e-05	4.6e-05	4.1e-05
Pz	1.4e-04	2.5e-04	5.7e-04	4.5e-04	4.5e-04	3.9e-04	3.6e-04
Ps	1.4e-02	4.4e-03	9.9e-04	9.9e-04	2.2e-03	1.8e-03	1.7e-03
T	0:59	1:11	1:21	1:40	1:13	1:14	1:23
I	0:07	0:14	0:27	0:34	0:16	0:22	0:25
E	0:52	0:57	0:54	1:06	0:57	0:52	0:58
mean_E	0.206	0.227	0.208	0.259	0.219	0.223	0.199
h	0:58:27	1:37:57	0:14:52	0:15:10	0:03:32	0:18:32	1:06:56
m	1:24:20	2:21:19	0:21:26	0:21:53	0:05:06	0:26:44	1:36:34

gene	YJL001W	YOR157C	YER094C	YER012W	YPR103W	YBL041W	YFR050C
subunit	beta-1	beta-2	beta-3	beta-4	beta-5	beta-6	beta-7
L	216	262	206	199	288	242	267
x	16.64	3.99	22.61	4.09	14.08	7.15	9.41
B	11185	2581	2468	2674	2670	1657	3549
g	0.86	4.32	0.61	3.02	0.83	2.21	1.42
w	1.86	11.31	1.26	6.01	2.39	5.35	3.80
P	3.9e-05	1.8e-04	2.7e-05	1.4e-04	4.3e-05	1.0e-04	6.7e-05
Pz	4.6e-04	1.1e-04	6.3e-04	1.1e-04	3.9e-04	2.0e-04	2.6e-04
Ps	1.3e-03	2.6e-02	6.6e-04	1.9e-02	1.7e-03	7.9e-03	3.9e-03
T	1:12	1:05	1:22	0:49	1:19	1:01	1:11
I	0:25	0:05	0:36	0:07	0:23	0:10	0:15
E	0:47	1:00	0:46	0:42	0:56	0:51	0:56
mean_E	0.218	0.228	0.223	0.209	0.194	0.213	0.212
h	3:16:42	0:39:32	0:45:47	0:52:24	0:51:19	0:25:48	1:04:52
m	4:43:48	0:57:03	1:06:03	1:15:36	1:14:02	0:37:33	1:33:34
