# Supplementary material for: A Comprehensive, Quantitative, and Genome-Wide Model of Translation
Source: PLoS Comput Biol. 2010 Jul 29;6(7):e1000865. doi: 10.1371/journal.pcbi.1000865 (PMC2912337; doi:10.1371/journal.pcbi.1000865)
Supplement: Table S4 — Decoding specificities of yeast tRNAs and calculated values of the model parameters for particular codons. (0.02 MB PDF) [file pcbi.1000865.s006.pdf]

Table S4: Decoding specificities of yeast tRNAs (determined by [63]) and calculated values of the model parameters for particular codons. Column descriptions: (1) tRNA number; (2) codons recognised by a tRNA molecule; (3) tRNA anticodon; (4) coded amino acid; (5) number of tRNA genes; (6) number of tRNA genes divided by 274 - the total number of tRNA genes in the yeast genome; (7) diffusion coefficient of a aa-tRNA molecule in 10^{-11} m^2/sec (taken from [60]); (8) the number of tRNA molecules in a yeast cell; (9) characteristic time τ of a aa-tRNA molecule moving from one occupation site of a cell to the other in 10^{-5} seconds; and (10) time t that elapses before the arrival of a aa-tRNA molecule to the vacant A site of a ribosome in seconds.

nr	codon	anti-codon	amino-acid	nr genes	fr	diff coeff	mol cell	τ	t
1	GCA,GCG	UGC	Ala	5	0.018	0.2571	51437	1.3588	0.0037
2	GCU,GCC	IGC	Ala	11	0.040	0.2571	113163	1.3588	0.0017
3	AGA	UCU	Arg	11	0.040	0.2568	113163	1.3604	0.0017
4	AGG	CCU	Arg	1	0.004	0.2568	10289	1.3604	0.0183
5	CGG	CCG	Arg	1	0.004	0.2568	10289	1.3604	0.0183
6	CGU,CGC,CGA	ICG	Arg	6	0.022	0.2568	61726	1.3604	0.0031
7	AAU,AAC	GUU	Asn	10	0.036	0.2570	102875	1.3593	0.0018
8	GAU,GAC	GUC	Asp	15	0.055	0.2570	154315	1.3593	0.0012
9	UGU,UGC	GCA	Cys	4	0.015	0.2570	41152	1.3593	0.0046
10	CAA	UUG	Gln	9	0.033	0.2569	92589	1.3598	0.0020
11	CAG	CUG	Gln	1	0.004	0.2569	10289	1.3598	0.0183
12	GAA	UUC	Glu	14	0.051	0.2569	144026	1.3598	0.0013
13	GAG	CUC	Glu	2	0.007	0.2569	20574	1.3598	0.0091
14	GGA	UCC	Gly	3	0.011	0.2572	30863	1.3583	0.0061
5	GGG	CCC	Gly	2	0.007	0.2572	20574	1.3583	0.0091
16	GGU,GGC	GCC	Gly	16	0.058	0.2572	164601	1.3583	0.0011
17	CAU,CAC	GUG	His	7	0.026	0.2569	72012	1.3598	0.0026
18	AUA	UAU	Ile	2	0.007	0.2570	20574	1.3593	0.0091
19	AUC,AUU	IAU	Ile	13	0.047	0.2570	133738	1.3593	0.0014
20	CUA,CUG	UAG	Leu	3	0.011	0.2570	30863	1.3593	0.0061
21	CUC,CUU	GAG	Leu	1	0.004	0.2570	10289	1.3593	0.0183
22	UUA	UAA	Leu	7	0.026	0.2570	72012	1.3593	0.0026
23	UUG	CAA	Leu	10	0.036	0.2570	102875	1.3593	0.0018
24	AAA	UUU	Lys	7	0.026	0.2569	72012	1.3598	0.0026
25	AAG	CUU	Lys	14	0.051	0.2569	144026	1.3598	0.0013
26	AUG	CAU	Met	5	0.018	0.2569	51437	1.3598	0.0037
27	UUU,UUC	GAA	Phe	10	0.036	0.2568	102875	1.3604	0.0018
28	CCA,CCG	UGG	Pro	10	0.036	0.2570	102875	1.3593	0.0018
29	CCU,CCC	IGG	Pro	2	0.007	0.2570	20574	1.3593	0.0091
30	AGU,AGC	GCU	Ser	4	0.015	0.2571	41152	1.3588	0.0046
31	UCA	UGA	Ser	3	0.011	0.2571	30863	1.3588	0.0061
32	UCG	CGA	Ser	1	0.004	0.2571	10289	1.3588	0.0183
33	UCU,UCC	IGA	Ser	11	0.040	0.2571	113163	1.3588	0.0017
34	ACA	UGU	Thr	4	0.015	0.2570	41152	1.3593	0.0046
35	ACG	CGU	Thr	1	0.004	0.2570	10289	1.3593	0.0183
36	ACU,ACC	IGU	Thr	11	0.040	0.2570	113163	1.3593	0.0017
37	UGG	CCA	Trp	6	0.022	0.2567	61726	1.3609	0.0031
38	UAU,UAC	GUA	Tyr	8	0.029	0.2568	82300	1.3604	0.0023
39	GUA	UAC	Val	2	0.007	0.2570	20574	1.3593	0.0091
40	GUG	CAC	Val	2	0.007	0.2570	20574	1.3593	0.0091
41	GUU,GUC	IAC	Val	14	0.051	0.2570	144026	1.3593	0.0013
